# Relative Deprivation and Moral Disengagement as Serial Mediators Between Cyberbullying Victimization and Psychological Distress Symptoms Among Victim-Only Five-Year Higher Vocational College Students

**DOI:** 10.3390/bs16060915

**Published:** 2026-06-03

**Authors:** Wei Song, Jingxin Wang

**Affiliations:** 1 Key Research Base of Humanities and Social Sciences of the Ministry of Education, Academy of Psychology and Behavior, Tianjin Normal University, Tianjin 300387, China; songwei@fjwzy.edu.cn; 2 Faculty of Psychology, Tianjin Normal University, Tianjin 300387, China; 3 Publicity Department, Fujian Health College, Fuzhou 350101, China; 4 Tianjin Key Laboratory of Student Mental Health and Intelligence Assessment, Tianjin 300387, China

**Keywords:** cyberbullying victimization, relative deprivation, moral disengagement, psychological distress symptoms, five-year higher vocational college students

## Abstract

Cyberbullying victimization is a public health concern associated with adolescents’ psychological distress symptoms. This cross-sectional study examined whether relative deprivation and moral disengagement were statistically associated with the relationship between cyberbullying victimization and psychological distress symptoms among victim-only five-year higher vocational college students. Among 4290 valid respondents, 1419 students reported at least one cyberbullying victimization experience. Because the present study focused on victimization without concurrent perpetration, 1107 victim-only students were included in the primary analysis. Participants completed self-report measures of cyberbullying victimization, relative deprivation, moral disengagement, and psychological distress symptoms. After controlling for gender and age, cyberbullying victimization was positively associated with psychological distress symptoms. The bootstrap results indicated significant indirect associations through relative deprivation, through moral disengagement, and through the serial pathway from relative deprivation to moral disengagement. These findings suggest that relative deprivation and moral disengagement are statistically linked to the association between cyberbullying victimization and psychological distress symptoms among victim-only vocational students. Given the cross-sectional self-report design, the mediation findings should be interpreted as evidence of statistical associations rather than temporal ordering or causal mechanisms.

## 1. Introduction

With the rapid development of internet technology and the widespread use of social media platforms, cyberbullying has become a significant social problem affecting adolescents worldwide. Cyberbullying victimization can be broadly understood as being subjected to aggressive or bullying behavior directed at an individual or group through electronic means (Kasturiratna et al., 2025). Unlike traditional bullying, repetition in online contexts may operate through repeated viewing, sharing, forwarding, or archiving of a single harmful post, image, or message, even when the perpetrator does not repeatedly act (Tokunaga, 2010). This conceptualization is adopted in the present study because the Revised Cyberbullying Inventory covers diverse forms of online victimization, including threatening messages, exclusion, information theft, and disclosure of private conversations. Previous research has shown that cyberbullying victimization is associated with psychological distress symptoms such as depression, anxiety, loneliness, low self-esteem, and suicidal ideation (Chu et al., 2018; Kowalski et al., 2014).

Five-year higher vocational college students represent a distinctive educational population in China’s vocational education system. These students enter vocational education directly after completing junior high school and undergo five years of continuous training (Liu, 2023). Compared with students in regular academic tracks, five-year vocational students may face different educational trajectories, academic experiences, and psychosocial adjustment demands (Bao, 2020). These characteristics make it important to examine cyberbullying victimization and psychological distress symptoms in this specific population. However, research specifically focusing on five-year higher vocational college students remains scarce. Thus, the present study aimed to examine the psychological mechanisms underlying the association between cyberbullying victimization and psychological distress symptoms among five-year higher vocational college students.

### 1.1. Theoretical Framework

This study integrates social cognitive theory and social conflict theory to examine the mechanisms linking cyberbullying victimization to psychological distress symptoms. Social cognitive theory, proposed by Bandura (2001), emphasizes that individuals learn behaviors through observation and imitation within social interactions. In the context of cyberbullying victimization, individuals not only suffer direct attacks but also learn negative self-perceptions and threat appraisals through repeated exposure to hostile online environments. Moral disengagement, as a key cognitive mechanism within social cognitive theory, refers to the process by which individuals cognitively reconstruct harmful behaviors to make them appear less damaging, thereby reducing self-sanction and guilt (Bandura, 2014).

Social conflict theory, on the other hand, emphasizes that social problems often stem from structural inequalities and unfair resource distribution (Coser, 1967). Relative deprivation, a core concept in this theory, refers to the subjective perception of being disadvantaged compared to others, which can generate negative emotions such as anger, resentment, and hostility (Smith et al., 2012). For cyberbullying victims, experiencing online harassment may trigger feelings of relative deprivation, as they perceive themselves as unfairly targeted and disadvantaged compared to their peers.

### 1.2. Cyberbullying Victimization and Psychological Distress Symptoms

Psychological distress symptoms refer to a broad set of negative emotional states, including depressive symptoms, anxiety symptoms, stress, and general emotional discomfort (Lei et al., 2025; Song et al., 2025; H. Zhang et al., 2024). Previous research has documented significant associations between cyberbullying victimization and internalizing problems (Tsitsika et al., 2015). For example, meta-analytic and empirical studies have shown that cyberbullying victimization is positively associated with depression, anxiety, stress, loneliness, and related psychological distress symptoms (Chu et al., 2018; Kowalski et al., 2014). These findings suggest that cyberbullying victimization may be an important risk-related correlate of psychological distress symptoms among adolescents.

Therefore, based on previous findings and theoretical perspectives, our first hypothesis predicted that cyberbullying victimization would be positively associated with psychological distress symptoms among five-year higher vocational college students.

### 1.3. The Mediating Role of Relative Deprivation

Relative deprivation refers to the subjective feeling of being disadvantaged relative to others, arising from social comparisons that reveal discrepancies between one’s actual and expected situations (Smith et al., 2012). According to social conflict theory, when individuals perceive themselves as unfairly deprived of resources or opportunities, they may experience negative emotions that can lead to various psychological and behavioral outcomes (Coser, 1967).

Cyberbullying victimization may be associated with individuals’ relative deprivation by damaging their perceived social status and sense of fairness. Online victimization often occurs in peer contexts, and the public nature of digital aggression may make victims feel socially inferior or unjustly targeted. This perceived disadvantage can elicit frustration, resentment, helplessness, and negative self-perception. In turn, relative deprivation may be linked to psychological distress symptoms. Individuals who feel unfairly deprived may be more likely to engage in negative rumination, interpret social events pessimistically, and experience emotional exhaustion (J. Zhang et al., 2022; Chang et al., 2025). Thus, relative deprivation may represent a possible explanatory process statistically linking cyberbullying victimization to psychological distress symptoms. Consequently, our second hypothesis predicted that cyberbullying victimization would be indirectly associated with psychological distress symptoms through relative deprivation.

### 1.4. The Mediating Role of Moral Disengagement

Moral disengagement refers to a set of cognitive mechanisms through which individuals deactivate internal moral standards and reconstruct harmful behavior as acceptable, less damaging, or justified, thereby reducing self-sanction, guilt, and emotional discomfort (Bandura, 2014; Bandura et al., 1996). According to Bandura’s theory, moral disengagement involves several mechanisms, including moral justification, euphemistic labeling, advantageous comparison, displacement of responsibility, diffusion of responsibility, distortion of consequences, dehumanization, and attribution of blame. These mechanisms allow individuals to reinterpret harmful conduct, minimize its consequences, or shift responsibility away from the self, thereby weakening the moral self-regulatory processes that normally inhibit inappropriate behavior (Bandura, 2014).

While moral disengagement has been primarily studied as a predictor of aggressive behavior, recent research has examined its role in the victimization process. Previous studies have suggested that bullying victimization is associated with higher levels of moral disengagement, which is also related to psychological distress (Xia & Sha, 2021; J. Wang et al., 2018). Victims may use moral disengagement to rationalize their suffering or to justify potential retaliatory behaviors. Therefore, moral disengagement may be a possible explanation for the psychological distress symptoms experienced by victims of cyberbullying. Based on the above theoretical and empirical evidence, the present study proposes the following hypothesis: H3: Cyberbullying victimization will be indirectly associated with psychological distress symptoms through moral disengagement.

### 1.5. The Sequential Mediation Model

Relative deprivation and moral disengagement may not only function as independent mediators but may also operate in a sequential manner. Five-year higher vocational college students who experience cyberbullying victimization may report stronger feelings of relative deprivation because victimization may be perceived as unfair treatment, social disadvantage, or deprivation of respect and support. This sense of unfairness may be associated with moral disengagement. Students who perceive that they have been unjustly harmed may be more likely to justify negative reactions, externalize responsibility, or show reduced sensitivity to moral standards. In other words, relative deprivation may provide a cognitive-emotional basis for moral disengagement.

This sequential pathway is theoretically plausible because perceived injustice may be linked to maladaptive cognitive responses. Individuals who feel relatively deprived may be more likely to interpret social interactions through a hostile or unfairness-oriented lens (Coser, 1967; Smith et al., 2012). Such interpretations can weaken moral self-regulation and increase the use of disengagement mechanisms (Bandura, 2001, 2014). Higher moral disengagement may also be associated with greater difficulty in processing victimization experiences adaptively and with higher psychological distress symptoms (J. Wang et al., 2018; Xia & Sha, 2021). Therefore, cyberbullying victimization may be associated with psychological distress symptoms not only through relative deprivation or moral disengagement separately but also through the sequential pathway from relative deprivation to moral disengagement.

Based on the above theoretical and empirical considerations, the present study constructed a sequential mediation model to examine the association between cyberbullying victimization and psychological distress symptoms among five-year higher vocational college students, which may help clarify the psychological correlates and possible explanatory processes through which cyberbullying victimization is statistically associated with psychological distress symptoms among five-year higher vocational college students.

### 1.6. Current Study

Based on the theoretical framework and literature review, this study aims to examine the chain mediating role of relative deprivation and moral disengagement in the relationship between cyberbullying victimization and psychological distress symptoms among five-year higher vocational college students. The following hypotheses are proposed:

**Hypothesis** **1.** *Cyberbullying victimization would be positively associated with psychological distress symptoms among five-year higher vocational college students.*

**Hypothesis** **2.** *Relative deprivation would show a specific indirect association between cyberbullying victimization and psychological distress symptoms after accounting for moral disengagement.*

**Hypothesis** **3.** *Moral disengagement would show a specific indirect association between cyberbullying victimization and psychological distress symptoms after accounting for relative deprivation.*

**Hypothesis** **4.** *Cyberbullying victimization would be indirectly associated with psychological distress symptoms through the serial pathway of relative deprivation and moral disengagement.*

## 2. Materials and Methods

### 2.1. Participants and Procedure

This study employed a cross-sectional survey design. A total of 4989 five-year vocational students were invited to participate in the study. After informed consent was obtained, participants completed a set of anonymous self-report questionnaires assessing cyberbullying victimization, cyberbullying perpetration, and demographic information. After excluding incomplete questionnaires and patterned responses, 4290 valid responses were retained. Invalid patterned responses were defined as highly repetitive identical response patterns across multiple questionnaire items, together with invalid responses to validity-check items, suggesting inattentive or unreliable responding.

**Figure 1:**
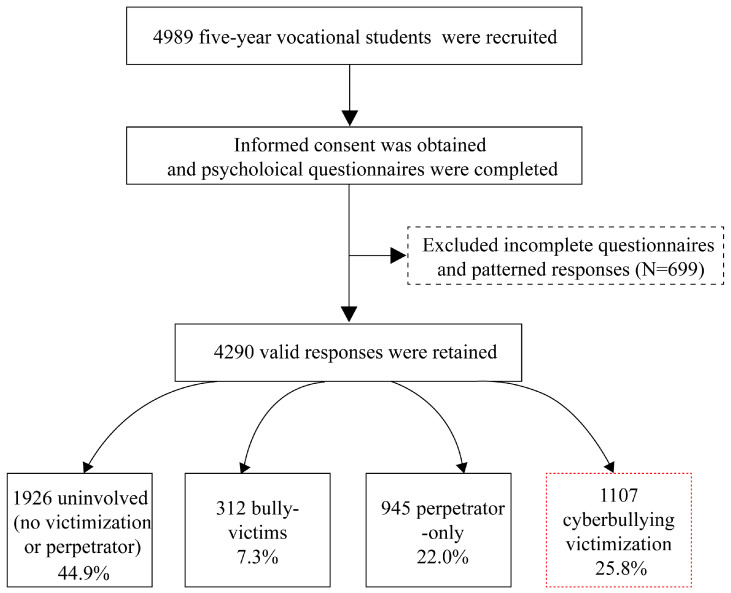
Flow diagram of participant recruitment and classification of cyberbullying involvement.

Cyberbullying victimization and cyberbullying perpetration were assessed using the corresponding subscales of the Revised Cyberbullying Inventory. Each item was rated on a 4-point scale from 1 = “Never” to 4 = “More than 3 times.” Cyberbullying victimization and perpetration status were classified based on item-level responses. Participants were classified as having experienced cyberbullying victimization if they reported any victimization behavior at least once. Similarly, participants were classified as having engaged in cyberbullying perpetration if they reported any perpetration behavior at least once (Chu & Fan, 2017). In this study, the criterion of cyberbullying victimization was equivalent to a total cyberbullying victimization score greater than 14. Among the 4290 valid respondents, 1926 students reported neither cyberbullying perpetration nor cyberbullying victimization, 945 students reported cyberbullying perpetration only, 1107 students reported cyberbullying victimization only, and 312 students were classified as bully-victims because they reported both cyberbullying perpetration and cyberbullying victimization. Because the present study focused on the psychological correlates of cyberbullying victimization without concurrent cyberbullying perpetration, only the 1107 victim-only students were included in the primary analysis. The process of participant recruitment and classification of cyberbullying involvement is shown in Figure 1.

Demographic and internet-use characteristics of the final analytic sample are presented in Table 1. The study was conducted in accordance with the Declaration of Helsinki and approved by the Institutional Review Board of the authors’ institution. Informed consent was obtained from all participants before data collection. Data were collected through online questionnaires administered during regular class hours. Participants completed the questionnaires anonymously, and all responses were kept confidential.

### 2.2. Measures

#### 2.2.1. Demographic Questionnaire

Considering the overall questionnaire structure and the characteristics of the study population, several demographic variables were selected to form the basic information section. This section included students’ gender, age, only-child status, residence, and parents’ educational background. In addition, given the focus of the present study on cyberbullying victimization, internet-use-related information was also collected, including access to digital devices and daily duration of internet use.

#### 2.2.2. Cyberbullying Victimization

Cyberbullying victimization was measured using the Cyberbullying Victimization Subscale of the Revised Cyberbullying Inventory (RCBI) (Topcu & Erdur-Baker, 2010). The Chinese version of this scale was adapted by Chu and Fan (Chu & Fan, 2017). The subscale consists of 14 items assessing various forms of cyberbullying victimization, including having personal information stolen, receiving threatening messages, being excluded online, and having private conversations shared without permission. Participants rated the frequency of each experience on a 4-point Likert scale (1 = Never, 4 = More than 3 times). Higher scores indicate greater levels of cyberbullying victimization. In this study, Cronbach’s alpha coefficient for this scale was 0.849.

#### 2.2.3. Relative Deprivation

Relative deprivation was measured using the Relative Deprivation Scale developed by Ma (2012). This scale consists of 4 items assessing individuals’ perceptions of unfair disadvantage compared to their efforts and others around them (e.g., “Compared to my efforts and contributions, my life should be better than it is now”). Items were rated on a 6-point Likert scale (1 = Strongly disagree, 6 = Strongly agree). Higher scores indicate stronger feelings of relative deprivation. Cronbach’s alpha coefficient for this scale in the current study was 0.799.

#### 2.2.4. Moral Disengagement

Moral disengagement was assessed using the Moral Disengagement Scale developed by Caprara et al. (2009) and adapted for Chinese populations by X. Wang et al. (2013). The scale comprises 32 items across eight dimensions: moral justification, advantageous comparison, euphemistic labeling, displacement of responsibility, diffusion of responsibility, distorting consequences, dehumanization, and attribution of blame. Participants rated their agreement with each statement on a 5-point Likert scale (1 = Completely disagree, 5 = Completely agree). Higher scores indicate higher levels of moral disengagement. In this study, Cronbach’s alpha coefficient was 0.962.

#### 2.2.5. Psychological Distress Symptoms

Psychological distress symptoms were assessed using the Chinese version of the Brief Symptom Inventory-18 (BSI-18) (Derogatis, 2001). The BSI-18 includes 18 items covering three symptom dimensions: somatization, depression, and anxiety with each dimension containing six items. Participants rated the severity of each symptom during the past week on a 5-point Likert scale ranging from 1 = “Not at all” to 5 = “Extremely.” In the present study, the total BSI-18 score was used as a continuous indicator of overall psychological distress, with higher scores indicating higher levels of psychological distress symptoms. Previous studies have examined the factor structure, internal consistency, and measurement invariance of the Chinese BSI-18 in Chinese samples, supporting its structural validity and good reliability (Geng et al., 2022). The scale has also been used to assess psychological distress symptoms among Chinese college students and adolescents (Lei et al., 2025; Song et al., 2025; H. Zhang et al., 2024). In the present study, Cronbach’s alpha coefficients for the total scale and the three subscales were 0.949, 0.847, 0.889, and 0.911, respectively. Clinical cut-off points were not applied, and the BSI-18 was not used for diagnostic classification in the present study.

### 2.3. Data Analysis

Data analysis was conducted using SPSS 24.0 (IBM Corporation, Armonk, NY, USA) and the PROCESS macro developed by Hayes (2017). PROCESS was specifically designed for testing complex models, including sequential mediation models using the bias-corrected percentile bootstrap method, and has been widely used in previous studies (Guo et al., 2018; Zhao et al., 2025). All continuous study variables were standardized before the mediation analyses. Based on previous literature, gender and age were included as covariates in the analyses (Li et al., 2021; Lu et al., 2019; H. Zhang et al., 2024). First, descriptive statistics were calculated for all study variables, and Pearson correlation analyses were performed to examine the associations among cyberbullying victimization, relative deprivation, moral disengagement, and psychological distress symptoms.

Second, a sequential mediation analysis was conducted using Model 6 of the PROCESS macro to examine whether relative deprivation and moral disengagement were statistically associated with the relationship between cyberbullying victimization and psychological distress symptoms. The significance of indirect effects was tested using the bias-corrected percentile bootstrap method with 5000 resamples. An indirect effect was considered statistically significant when the 95% bootstrap confidence interval did not include zero. The significance level for all analyses was set at *p* < 0.05.

## 3. Results

### 3.1. Common Method Bias and Preliminary Analyses

To examine for common method bias, we performed a Harman one-way factorial test. The results showed that the first unrotated factor explained 18.18% of the total variance, below the commonly used 40% threshold (Podsakoff et al., 2003). Furthermore, we performed variance inflation factor (VIF) analysis to assess multicollinearity among the predictors. All VIF values were below 5, indicating the absence of multicollinearity (Hair et al., 2019). These results suggest that no single factor dominated the variance. However, Harman’s single-factor test is limited and cannot rule out common method variance, particularly because all variables were assessed using self-report questionnaires at one time point. Therefore, common method variance should still be considered when interpreting the findings.

### 3.2. Descriptive Statistics and Correlations

Descriptive statistics and Pearson correlation results are presented in Table 2. According to the correlation analysis, all the main variables showed significantly positive correlations with each other. Specifically, cyberbullying victimization was positively correlated with relative deprivation (r = 0.14, *p* < 0.01), moral disengagement (r = 0.16, *p* < 0.01), and psychological distress symptoms (r = 0.26, *p* < 0.01), indicating that students who reported more severe cyberbullying victimization tended to report higher levels of relative deprivation, moral disengagement, and psychological distress symptoms. In addition, both relative deprivation and moral disengagement were positively correlated with psychological distress symptoms (r = 0.33, *p* < 0.01; r = 0.27, *p* < 0.01), suggesting that students with higher relative deprivation and moral disengagement tended to report more psychological distress symptoms. Relative deprivation was also positively correlated with moral disengagement (r = 0.55, *p* < 0.01). These results supported Hypothesis 1 and provided a preliminary basis for Hypothesis 2 to Hypothesis 4.

### 3.3. Sequential Mediation Analysis

To clarify the associations among cyberbullying victimization, relative deprivation, moral disengagement and psychological distress symptoms, all study variables were standardized, and PROCESS Model 6 was used to test the sequential mediation model. As shown in Table 3, cyberbullying victimization was positively associated with relative deprivation (β = 0.137, t = 4.59, *p* < 0.001), and the model explained 1.9% of the variance in relative deprivation. In the model predicting moral disengagement, cyberbullying victimization was positively associated with moral disengagement (β = 0.083, t = 3.30, *p* < 0.01), and relative deprivation was also positively associated with moral disengagement (β = 0.538, t = 21.39, *p* < 0.001). This model explained 31.7% of the variance in moral disengagement. In the final model, cyberbullying victimization remained positively associated with psychological distress symptoms (β = 0.218, t = 7.84, *p* < 0.001). Relative deprivation (β = 0.244, t = 7.45, *p* < 0.001) and moral disengagement (β = 0.106, t = 3.20, *p* < 0.01) were also positively associated with psychological distress symptoms. This model explained 18.1% of the variance in psychological distress symptoms. These results provide regression-based support for further testing the indirect effects of relative deprivation and moral disengagement.

**Figure 2:**
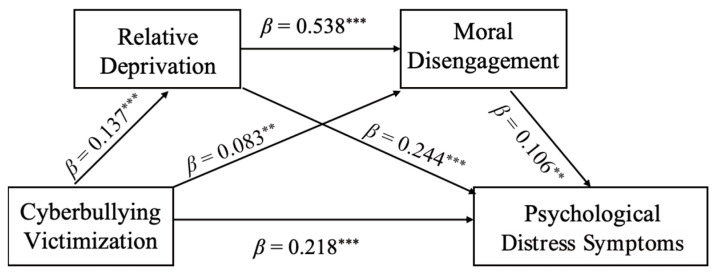
Chain mediation model of cyberbullying victimization, relative deprivation, moral disengagement, and psychological distress symptoms. ** *p* < 0.01; *** *p* < 0.001.

Bootstrap results are presented in Table 4. The total effect of cyberbullying victimization on psychological distress symptoms was significant (effect = 0.268, 95% CI [0.174, 0.365]). After relative deprivation and moral disengagement were included in the model, the direct effect remained significant (effect = 0.218, 95% CI [0.130, 0.311]), accounting for 81.3% of the total effect. The total indirect effect was also significant (effect = 0.050, 95% CI [0.028, 0.076]), accounting for 18.7% of the total effect. Specifically, the indirect association through relative deprivation was significant (effect = 0.033, 95% CI [0.015, 0.057]), accounting for 12.5% of the total effect. The indirect association through moral disengagement was also significant (effect = 0.009, 95% CI [0.001, 0.022]), accounting for 3.3% of the total effect. In addition, the serial indirect association through relative deprivation and moral disengagement was significant (effect = 0.008, 95% CI [0.001, 0.017]), accounting for 2.9% of the total effect. These findings supported Hypotheses 2 to 4, while the interpretation remains limited to cross-sectional statistical associations (see Table 4 and Figure 2).

## 4. Discussion

This study examined whether relative deprivation and moral disengagement were statistically associated with the relationship between cyberbullying victimization and psychological distress symptoms among victim-only five-year higher vocational college students. After controlling for gender and age, cyberbullying victimization was positively associated with psychological distress symptoms. The bootstrap results further showed significant indirect associations through relative deprivation, through moral disengagement, and through the serial pathway from relative deprivation to moral disengagement. These findings suggest that students who reported more severe cyberbullying victimization also tended to report higher levels of perceived unfair disadvantage and moral disengagement, which were in turn associated with greater psychological distress symptoms. However, because the present study used a cross-sectional self-report design, these findings should be interpreted as statistical associations rather than evidence of temporal ordering or causal mechanisms.

### 4.1. Mediating Effects of Relative Deprivation and Moral Disengagement

Consistent with the proposed model, both relative deprivation and moral disengagement showed significant indirect associations between cyberbullying victimization and psychological distress symptoms. The indirect association through relative deprivation accounted for 12.5% of the total effect, whereas the indirect association through moral disengagement accounted for 3.3%. This pattern suggests that relative deprivation may be a more prominent psychological correlate than moral disengagement in explaining why cyberbullying victimization is associated with psychological distress symptoms among victim-only students.

The mediating role of relative deprivation is consistent with social conflict theory, which emphasizes the psychological consequences of perceived unfairness and disadvantage (Coser, 1967; Smith et al., 2012). Cyberbullying victimization may make students feel unfairly targeted, socially disadvantaged, or deprived of the safe online environment enjoyed by their peers. These perceptions may be particularly salient among five-year higher vocational college students, who are in a developmental period marked by sensitivity to peer evaluation and social comparison. When students interpret online victimization as unfair treatment or social exclusion, they may experience resentment, helplessness, frustration, and negative self-evaluation. These emotional and cognitive responses may be linked to higher levels of anxiety, depression, somatic complaints, and other psychological distress symptoms. This finding is also consistent with previous research showing that relative deprivation is associated with adverse psychological outcomes (J. Zhang et al., 2022; Chang et al., 2025).

The indirect association through moral disengagement was also statistically significant, although its effect size was relatively small. This result is consistent with social cognitive theory, which suggests that individuals’ social experiences are related to their cognitive appraisals and self-regulatory processes (Bandura, 2001, 2014). Although moral disengagement has often been examined as a predictor of aggressive behavior, the present findings suggest that it may also be relevant to victimization-related psychological adjustment. Students who experience cyberbullying victimization may endorse moral disengagement cognitions to rationalize their experiences, reduce emotional discomfort, or justify potential retaliatory responses. However, such cognitive patterns may not facilitate adaptive coping. Instead, they may maintain maladaptive interpretations of interpersonal harm and contribute to psychological distress symptoms. Nevertheless, given the modest effect size and the cross-sectional design, this pathway should be interpreted cautiously, and causal inferences are not warranted.

### 4.2. Chain Mediation Mechanism

The serial indirect association through relative deprivation and moral disengagement was significant, supporting the proposed sequential mediation model. Specifically, cyberbullying victimization was associated with higher relative deprivation, relative deprivation was associated with higher moral disengagement, and moral disengagement was associated with higher psychological distress symptoms. This finding suggests that perceived unfair disadvantage and moral cognition may operate jointly in the association between cyberbullying victimization and psychological distress symptoms.

One possible interpretation is that cyberbullying victimization is associated with a sense of unfairness or social disadvantage. Students who feel that they have been unjustly harmed may be more likely to interpret social interactions through a hostile or unfairness-oriented lens. This sense of relative deprivation may then be associated with moral disengagement, such as justifying negative reactions, minimizing the consequences of harmful online behavior, or attributing blame to others. Higher moral disengagement may then be linked to greater difficulty in processing victimization experiences adaptively, which may be associated with higher psychological distress symptoms. However, the serial pathway accounted for only 2.9% of the total effect. Therefore, although the chain mediation pathway was statistically significant, it should not be described as the dominant explanatory pathway. The direct association between cyberbullying victimization and psychological distress symptoms remained substantial, and the total indirect effect accounted for 18.7% of the total effect. These results indicate that relative deprivation and moral disengagement are relevant but partial correlates of psychological distress symptoms. Other factors, such as perceived social support, self-esteem, coping style, emotion regulation, school climate, and family support, may also play important roles and should be examined in future research.

In addition, alternative models remain plausible. Students with higher psychological distress symptoms may be more likely to perceive online interactions as threatening, unfair, or humiliating (Mathews & MacLeod, 2005; Miers et al., 2008). Moral disengagement may also influence how students interpret or report cyberbullying experiences (Bandura, 2014; Smith et al., 2012). Therefore, the present serial mediation model should be understood as a theoretically guided statistical model.

### 4.3. Theoretical and Practical Implications

This study has several theoretical implications. First, it extends cyberbullying research by focusing on victim-only five-year higher vocational college students, a population that has received limited empirical attention. By excluding bully-victims from the primary analysis, the study reduced potential confounding from concurrent perpetration experiences and provided a more specific examination of psychological correlates among students who experienced victimization without reported perpetration. Second, the study integrates social conflict theory and social cognitive theory in a single statistical model. The significant serial association between the relative deprivation and moral disengagement suggests that perceived injustice and moral cognition may be jointly relevant to psychological distress symptoms. Third, the stronger indirect association through relative deprivation indicates that perceived unfair disadvantage may deserve greater theoretical attention in explaining the psychological adjustment of cyberbullying victims. Cyberbullying is not merely an interpersonal stressor; it may also be experienced as an unfair social experience that damages students’ sense of equality, respect, and belonging. This perspective may help future studies develop more precise models of cyberbullying-related psychological distress.

The findings also have practical implications. School-based cyberbullying prevention and counseling programs may benefit from addressing students’ perceptions of unfairness, humiliation, and social disadvantage after victimization. Counselors and educators could help victims develop more balanced social comparisons, reduce persistent rumination about unfair treatment, and rebuild a sense of interpersonal safety.

### 4.4. Limitations and Future Directions

Several limitations should be acknowledged. First, the cross-sectional design precludes causal and temporal inferences. Longitudinal studies are needed to further clarify the temporal and reciprocal relationships among these variables. Second, all variables were measured using self-report questionnaires, which may introduce common method variance and response bias. Future research should incorporate multiple sources of data, such as peer reports, teacher reports, behavioral records, or school cyberbullying incident reports. Third, the primary analysis focused on victim-only students and excluded bully-victims. This decision helped isolate the psychological correlates of victimization without concurrent perpetration, but it limits the generalizability of the findings to students with mixed cyberbullying involvement. Future studies should compare victim-only students, bully-victims, perpetrator-only students, and uninvolved students. Fourth, the sample consisted of five-year higher vocational college students in China, so the findings may not generalize to students in other educational or cultural contexts. Finally, the model included only relative deprivation and moral disengagement. Given that the direct effect of cyberbullying victimization remained significant, future studies should examine additional factors such as self-esteem, social support, emotion regulation, coping style, school belonging, family functioning, and teacher support. Future studies should examine whether the findings remain robust after controlling for additional sociodemographic variables, such as only-child status, residence, parental education, and family socioeconomic status.

## 5. Conclusions

In summary, this study found that cyberbullying victimization was positively associated with psychological distress symptoms among victim-only five-year higher vocational college students. Relative deprivation and moral disengagement each showed significant indirect associations, and the serial pathway from relative deprivation to moral disengagement was also significant. The findings suggest that perceived unfair disadvantage and moral disengagement are relevant cognitive and emotional correlates of psychological distress symptoms among cyberbullying victims. However, because of the cross-sectional self-report design, the results should be interpreted as statistical associations rather than causal mechanisms. Future longitudinal and intervention studies are needed to further clarify these relationships.

## Figures and Tables

**Table 1:** Demographic characteristics of the final analytic sample (N = 1107).

Characteristic	Category	N	%
Gender	Male	520	47.0
	Female	587	53.0
Only-child status	Only child	209	18.9
	Non-only child	898	81.1
Residence	Urban	538	48.6
	Rural	569	51.4
Father’s education	College level or above	139	12.6
	Below college level	968	87.4
Mother’s education	College level or above	101	9.1
	Below college level	1006	90.9
Allowed to bring mobile phone	Yes	1082	97.7
	No	25	2.3
Allowed to bring computer	Yes	1046	94.5
	No	61	5.5
Daily internet use	<1 h	54	4.9
	1–3 h	319	28.8
	3–5 h	472	42.6
	6–8 h	230	20.8
	≥9 h	32	2.9

**Table 2:** Descriptive statistics and correlations among study variables (N = 1107).

Variable	M ± SD	1	2	3	4	5	6
1. Gender	-	1					
2. Age	18.36 ± 0.90	−0.02	1				
3. Cyberbullying Victimization	17.17 ± 3.16	−0.07 *	−0.06	1			
4. Relative Deprivation	10.61 ± 4.09	0.00	−0.03	0.14 **	1		
5. Moral Disengagement	58.76 ± 19.88	−0.07 *	−0.07 *	0.16 **	0.55 **	1	
6. Psychological Distress Symptoms	25.13 ± 9.75	0.11 **	−0.05	0.26 **	0.33 **	0.27 **	1

Note. N = 1107. Gender was coded as 0 = male and 1 = female. * *p* < 0.05; ** *p* < 0.01. M = mean; SD = standard deviation.

**Table 3:** Regression analysis results (N = 1107).

Dependent Variable/Outcome	Independent Variable/Predictor	*R* ^2^	*F*	*β*	*t*
Relative Deprivation		0.019	7.28 **		
	Cyberbullying Victimization			0.137	4.59 ***
	Gender			0.026	0.43
	Age			−0.02	−0.61
Moral Disengagement		0.317	128.11 ***		
	Cyberbullying Victimization			0.083	3.30 **
	Relative Deprivation			0.538	21.39 ***
	Gender			−0.127	−2.53 *
	Age			−0.06	−2.15 *
Psychological Distress Symptoms		0.181	48.73 ***		
	Cyberbullying Victimization			0.218	7.84 ***
	Relative Deprivation			0.244	7.45 ***
	Moral Disengagement			0.106	3.20 **
	Gender			0.261	4.74 ***
	Age			−0.018	−0.60

Note. Standardized coefficients are reported. Gender and age were included as covariates. * *p* < 0.05; ** *p* < 0.01; *** *p* < 0.001.

**Table 4:** Bootstrap results of chain mediation effect (N = 1107).

Path	Effect	SE	LLCI	ULCI	Effect Ratio
Total Effect	0.268	0.049	0.174	0.365	100.0%
Direct Effect	0.218	0.047	0.130	0.311	81.3%
Total Indirect Effect	0.05	0.012	0.028	0.076	18.7%
CB → RD → PS	0.033	0.011	0.015	0.057	12.5%
CB → MD → PS	0.009	0.005	0.001	0.022	3.3%
CB → RD → MD → PS	0.008	0.004	0.001	0.017	2.9%

Note. CB = cyberbullying victimization; RD = relative deprivation; MD = moral disengagement; PS = psychological distress symptoms. Gender and age were included as covariates. Bootstrap resamples = 5000. SE = standard error. LLCI = lower limit confidence interval; ULCI = upper limit confidence interval. Confidence intervals are reported to three decimals.

## Data Availability

The data presented in this study are available on request from the corresponding author.

## References

[B1-behavsci-16-00915] Bandura A. (2001). Social cognitive theory: An agentic perspective. Annual Review of Psychology.

[B2-behavsci-16-00915] Bandura A. (2014). Social cognitive theory of moral thought and action. Handbook of moral behavior and development.

[B3-behavsci-16-00915] Bandura A., Barbaranelli C., Caprara G. V., Pastorelli C. (1996). Mechanisms of moral disengagement in the exercise of moral agency. Journal of Personality and Social Psychology.

[B4-behavsci-16-00915] Bao Y. (2020). A research on the current situation of academic self-efficacy in the five-year system higher vocational college students—Take C school of Jiangsu higher vocational college as an example. Journal of Hubei Open Vocational College.

[B5-behavsci-16-00915] Caprara G. V., Fida R., Vecchione M., Tramontano C., Barbaranelli C. (2009). Assessing civic moral disengagement: Dimensionality and construct validity. Personality and Individual Differences.

[B6-behavsci-16-00915] Chang L., Xu J., Zhao Y., Zhang H. (2025). Bullying victimization and problematic internet use among adolescents: The role of relative deprivation and anxiety. Journal of Genetic Psychology.

[B7-behavsci-16-00915] Chu X. W., Fan C. Y. (2017). Revision of the revised cyber bullying inventory among junior high school students. Chinese Journal of Clinical Psychology.

[B8-behavsci-16-00915] Chu X. W., Fan C. Y., Liu Q. Q., Zhou Z. K. (2018). Cyberbullying victimization and symptoms of depression and anxiety among Chinese adolescents: Examining hopelessness as a mediator and self-compassion as a moderator. Computers in Human Behavior.

[B9-behavsci-16-00915] Coser L. A. (1967). Continuities in the study of social conflict.

[B10-behavsci-16-00915] Derogatis L. R. (2001). BSI 18, brief symptom inventory 18: Administration, scoring and procedures manual.

[B11-behavsci-16-00915] Geng Y., Ni X., Wang Y., Fan J., Qian Y., Li X. (2022). Factor structure and measurement invariance of the brief symptom inventory-18 among Chinese adults. Frontiers in Psychology.

[B12-behavsci-16-00915] Guo L., Tian L., Huebner E. S. (2018). Family dysfunction and anxiety in adolescents: A moderated mediation model of self-esteem and perceived school stress. Journal of School Psychology.

[B13-behavsci-16-00915] Hair J. F., Black W. C., Babin B. J., Anderson R. E. (2019). Multivariate data analysis.

[B14-behavsci-16-00915] Hayes A. F. (2017). Introduction to mediation, moderation, and conditional process analysis: A regression-based approach.

[B15-behavsci-16-00915] Kasturiratna K. T. A. S., Hartanto A., Chen C. H. Y., Tong E. M. W., Majeed N. M. (2025). Umbrella review of meta-analyses on the risk factors, protective factors, consequences and interventions of cyberbullying victimization. Nature Human Behaviour.

[B16-behavsci-16-00915] Kowalski R. M., Giumetti G. W., Schroeder A. N., Lattanner M. R. (2014). Bullying in the digital age: A critical review and meta-analysis of cyberbullying research among youth. Psychological Bulletin.

[B17-behavsci-16-00915] Lei Y., Duan C., Shen K. (2025). Development and validation of the Chinese mental health value scale: A tool for culturally-informed psychological assessment. Frontiers in Psychology.

[B18-behavsci-16-00915] Li Y., Li J., Yang Z., Zhang J., Dong L., Wang F., Zhang J. (2021). Gender differences in anxiety, depression, and nursing needs among isolated coronavirus disease 2019 patients. Frontiers in Psychology.

[B19-behavsci-16-00915] Liu K. (2023). The contemporary value and future direction of five-year consistent higher vocational education in the context of building a strong education country. Chinese Vocational and Technical Education.

[B20-behavsci-16-00915] Lu Y., Alvarez A. N., Miller M. J. (2019). Measurement invariance of the Brief Symptom Inventory-18 (BSI-18) across Asian American ethnic, nativity, and gender groups. Asian American Journal of Psychology.

[B21-behavsci-16-00915] Ma A. (2012). Relative deprivation and social adaption: The role of mediator and moderator. Acta Psychologica Sinica.

[B22-behavsci-16-00915] Mathews A., MacLeod C. (2005). Cognitive vulnerability to emotional disorders. Annual Review of Clinical Psychology.

[B23-behavsci-16-00915] Miers A. C., Blöte A. W., Bögels S. M., Westenberg P. M. (2008). Interpretation bias and social anxiety in adolescents. Journal of Anxiety Disorders.

[B24-behavsci-16-00915] Podsakoff P. M., MacKenzie S. B., Lee J. Y., Podsakoff N. P. (2003). Common method biases in behavioral research: A critical review of the literature and recommended remedies. Journal of Applied Psychology.

[B25-behavsci-16-00915] Smith H. J., Pettigrew T. F., Pippin G. M., Bialosiewicz S. (2012). Relative deprivation: A theoretical and meta-analytic review. Personality and Social Psychology Review.

[B26-behavsci-16-00915] Song Y., Li H., Song Y., Song G., Su Q., Liu N., Zheng Z., Sun Y. (2025). Isolation, social support, and COVID-19-burnout among college students in a university in eastern China. Frontiers in Psychology.

[B27-behavsci-16-00915] Tokunaga R. S. (2010). Following you home from school: A critical review and synthesis of research on cyberbullying victimization. Computers in Human Behavior.

[B28-behavsci-16-00915] Topcu C., Erdur-Baker O. (2010). The revised cyber bullying inventory (RCBI): Validity and reliability studies. Procedia-Social and Behavioral Sciences.

[B29-behavsci-16-00915] Tsitsika A., Janikian M., Wójcik S., Makaruk K., Tzavela E., Tzavara C., Greydanus D., Merrick J., Richardson C. (2015). Cyberbullying victimization prevalence and associations with internalizing and externalizing problems among adolescents in six European countries. Computers in Human Behavior.

[B30-behavsci-16-00915] Wang J., Liu J., Wang F. (2018). Transformation from offline victim to online bully: The mediating role of moral disengagement and the strengthening effect of high self-esteem. Psychological Exploration.

[B31-behavsci-16-00915] Wang X., Yang J., Gao L. (2013). The Chinese version of civic moral disengament scale. Studies of Psychology and Behavior.

[B32-behavsci-16-00915] Xia P., Sha J. (2021). The impact of cyberbullying on adolescent aggressive behavior—The mediating role of moral disengagement. Journal of Chinese People’s Public Security University (Social Sciences Edition).

[B33-behavsci-16-00915] Zhang H., Chen C., Zhang L., Xue S., Tang W. (2024). The association between the deviation from balanced time perspective on adolescent pandemic mobile phone addiction: The moderating role of self-control and the mediating role of psychological distress. Frontiers in Psychology.

[B34-behavsci-16-00915] Zhang J., Gu J., Wang W. (2022). The relationship between bullying victimization and cyber aggression among college students: The mediating effects of relative deprivation and depression. Psychology Research and Behavior Management.

[B35-behavsci-16-00915] Zhao K., Liu Y., Shi Y., Bi D., Zhang C., Chen R., Jin Z. (2025). Mobile phone addiction and interpersonal problems among Chinese young adults: The mediating roles of social anxiety and loneliness. BMC Psychology.

